# Supplementary figures and images for: May predator body-size hamper furtive predation strategy by aphidophagous insects?
Source: PLoS One. 2021 Sep 2;16(9):e0256991. doi: 10.1371/journal.pone.0256991 (PMC8412377; doi:10.1371/journal.pone.0256991)

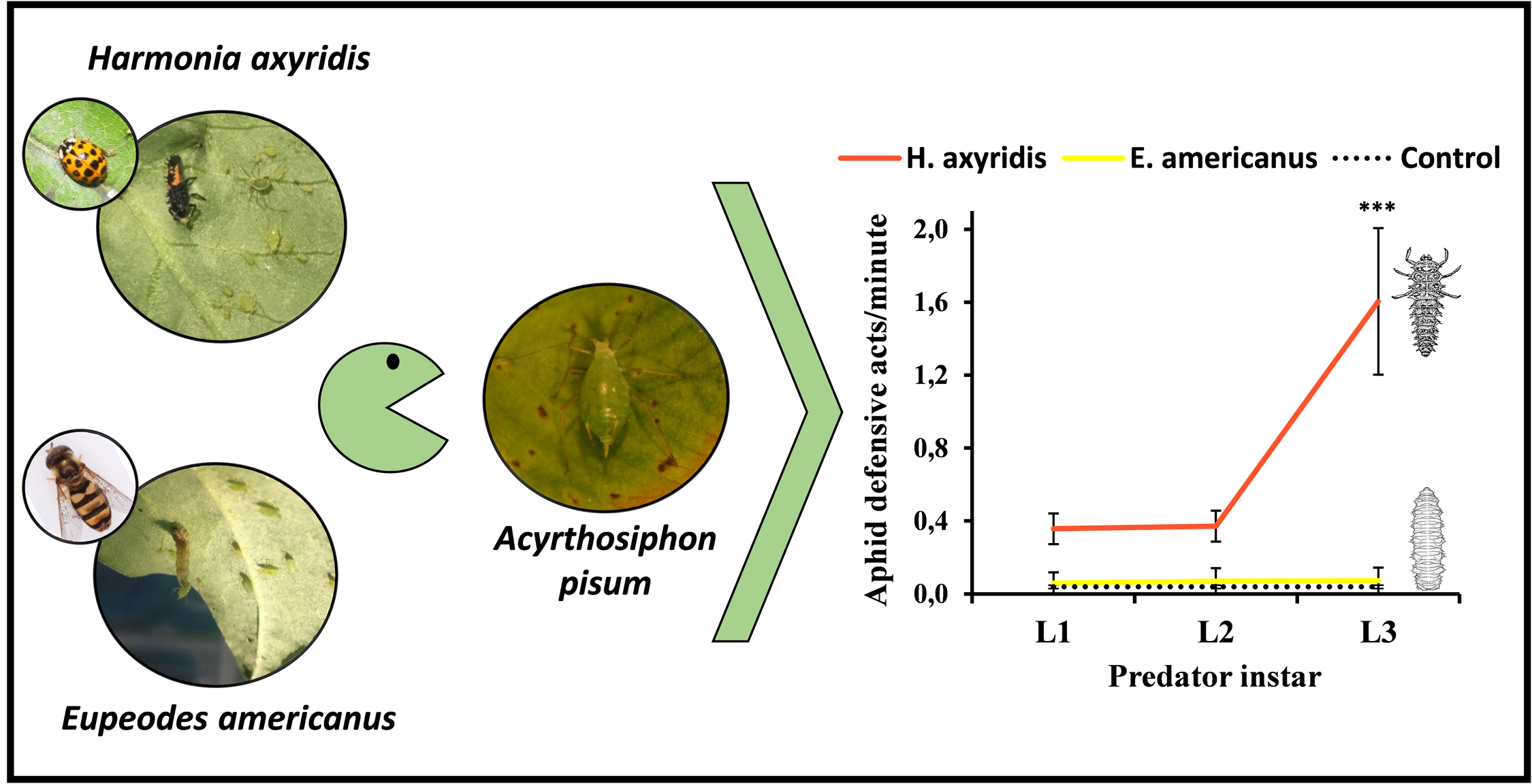

Supplement: S1 Fig — (TIF) [file pone.0256991.s001.tif]
